# The MELFO Study: A Multicenter, Prospective, Randomized Clinical Trial on the Effects of a Reduced Stage-Adjusted Follow-Up Schedule on Cutaneous Melanoma IB–IIC Patients—Results After 3 Years

**DOI:** 10.1245/s10434-019-07825-7

**Published:** 2019-09-18

**Authors:** Eric A. Deckers, Josette E. H. M. Hoekstra-Weebers, Samantha Damude, Anne Brecht Francken, Sylvia ter Meulen, Esther Bastiaannet, Harald J. Hoekstra

**Affiliations:** 1 Department of Surgical Oncology, University of Groningen, University Medical Center Groningen, Groningen, The Netherlands; 2 Wenckebach Institute, University of Groningen, University Medical Center Groningen, Groningen, The Netherlands; 3 Netherlands Comprehensive Cancer Organization, Utrecht, The Netherlands; 4 Department of Surgical Oncology, Isala Clinics, Zwolle, The Netherlands; 5 Skin-Melanoma Clinic, Netherlands Cancer Institute/Antoni van Leeuwenhoek, Amsterdam, The Netherlands; 6 Department of Surgical Oncology, Leiden University Medical Center, Leiden, The Netherlands

## Abstract

**Background:**

This study compares well-being, recurrences, and deaths of early-stage cutaneous melanoma patients in follow-up, as recommended in the Dutch guideline, with that of patients in a stage-adjusted reduced follow-up schedule, 3 years after diagnosis, as well as costs.

**Methods:**

Overall, 180 eligible pathological American Joint Committee on Cancer (AJCC) stage IB–IIC, sentinel node staged, melanoma patients (response rate = 87%, 48% male, median age 57 years), randomized into a conventional (CSG, *n* = 93) or experimental (ESG, *n* = 87) follow-up schedule group, completed patient-reported outcome measures (PROMs) at diagnosis (T1): State-Trait Anxiety Inventory–State version (STAI-S), Cancer Worry Scale (CWS), Impact of Event Scale (IES), and RAND-36 (Mental and Physical Component scales [PCS/MCS]). Three years later (T3), 110 patients (CSG, *n* = 56; ESG, *n* = 54) completed PROMs, while 42 declined (23%).

**Results:**

Repeated measures analyses of variance (ANOVAs) showed a significant group effect on the IES (*p* = 0.001) in favor of the ESG, and on the RAND-36 PCS (*p* = 0.02) favoring the CSG. Mean IES and CWS scores decreased significantly over time, while those on the RAND-36 MCS and PCS increased. Effect sizes were small. Twenty-five patients developed a recurrence or second primary melanoma, of whom 13 patients died within 3 years. Cox proportional hazards models showed no differences between groups in recurrence-free survival (hazard ratio [HR] 0.71 [0.32–1.58]; *p* = 0.400) and disease-free survival (HR 1.24 [0.42–3.71]; *p* = 0.690). Costs per patient after 3 years (computed for 77.3% of patients) were 39% lower in the ESG.

**Conclusion:**

These results seemingly support the notion that a stage-adjusted reduced follow-up schedule forms an appropriate, safe, and cost-effective alternative for pathological AJCC stage IB–IIC melanoma patients to the follow-up regimen as advised in the current melanoma guideline.

The worldwide incidence of cutaneous melanoma has increased over the past decade.^1^ In The Netherlands, the incidence of melanoma quadrupled between 1990 and 2018, from 1561 to 7046 new cases.^2^ However, the increase in mortality was lower. This rate doubled between 1990 and 2010, from 348 to 783 cases, but then stabilized. In 2017, 796 patients died of melanoma.^3^ Consequently, the prevalence of melanoma is increasing in The Netherlands.

Increasing prevalence results in a growing number of patients in follow-up. Most guidelines regarding follow-up schedules recommend at least 5-year, 10-year, or lifelong surveillance, which makes melanoma follow-up a burden in both time and financial costs.^4^,^5^ Additionally, patients are exposed to many outpatient clinic or general practitioner (GP) visits, which may result in emotional stress.^5^–^7^

Most of the recommendations in the current guidelines are based on recurrence risk, early detection, and, consequently, improved survival.^8^–^12^ Almost 90% of the recurrences occur in the first 3 years after primary diagnosis.^4^,^9^,^12^–^14^ Patients with a higher stage at primary diagnosis have a higher risk of recurrence, and the risk of recurrence after 10 years follow-up is low (2.4%).^6^,^7^,^10^,^15^

The lack of consensus in guidelines regarding the follow-up of cutaneous melanoma patients was the reason to initiate the melanoma follow-up study (MELFO). Preliminary 1-year results showed that a stage-adjusted, reduced follow-up schedule adversely affected neither patients’ well-being nor the number of recurrences or melanoma deaths, and that financial costs were lower compared with the conventional follow-up schedule recommended in the Dutch guideline.^16^

The aims of the present study were to examine comparability in (1) well-being and (2) the number and time of recurrences and deaths of early-staged melanoma patients who were subjected to the follow-up schedule advised in the Dutch guideline, as well as patients who received a stage-adjusted reduced follow-up schedule, 3 years after diagnosis. The hypotheses were that there would be no differences between the two groups in these outcomes and (3) that costs would be lower when patients were followed-up less frequently.

## Methods

### Study Design

Detailed methods of this multicenter, randomized clinical trial (NCT0108004), initiated by the Department of Surgical Oncology of the University Medical Center Groningen (UMCG), have been described previously.^16^ Participants were randomized into two groups: one group following the conventional schedule recommended in the Dutch Melanoma guideline, and one group whose follow-up was a stage-adjusted reduced schedule (Table 1). The primary endpoint was patients’ well-being. Secondary endpoints were recurrences, melanoma-related deaths, and costs.^16^

**Table 1:** Frequency of follow-up visits for the conventional follow-up schedule, as recommended by the Dutch Melanoma guideline, and a reduced and stage-adjusted experimental follow-up schedule^16^

Conventional follow-up schedule							Experimental follow-up schedule						
Years^a^	1	2	3	4	5	6–10	Years^a^	1	2	3	4	5	6–10
AJCC stage							AJCC stage						
IB	4	3	2	2	2		IB	1	1	1	1	1	1
IIA	4	3	2	2	2	1	IIA	2	2	1	1	1	1
IIB	4	3	2	2	2	1	IIB	3	3	2	1	1	1
IIC	4	3	2	2	2	1	IIC	3	3	2	1	1	1

*AJCC* American Joint Committee on Cancer, 7th edition

^a^Year after surgery for primary melanoma

### Patients and Procedure

Inclusion criteria were sentinel lymph node-negative melanoma patients with pathological American Joint on Cancer Committee (AJCC, 7th edition) stage IB–IIC, who had undergone surgery with a curative intent between 2006 and 2013. Patients aged < 18 or > 85 years, those not mastering the Dutch language sufficiently, and those who had another malignancy were excluded.

Eligible patients were randomized into the conventional (CSG) or experimental schedule group (ESG) after giving informed consent. The Netherlands Comprehensive Cancer Organization (IKNL) performed randomization and data management.

Patients completed questionnaires at study entry, which was shortly after diagnosis (T1), and at 1 (T2) and 3 years later (T3). Patients were excluded from T2 or T3 in cases of recurrence, a second primary, or when they had died. Clinicians provided follow-up information on all patients included at T1 during the 3 years of the study^16^ or until patients developed a recurrence, a second primary, or died. The present study focused on T1 and T3.

This study was approved by the Medical Ethics Committee of the UMCG (METc2004.127).

### Instruments

Patients answered questions on sex, age, level of education, relationship status, daily activities, and comorbidities at T1. They also answered questions on schedule satisfaction, frequency of self-inspection, and the number of melanoma-related GP visits at T1 and T3. Medical specialists provided diagnostic (primary melanoma site, Breslow thickness, ulceration, AJCC classification) and follow-up information (date of every outpatient visit, date and location of recurrence, date and cause of death).

Patients completed the following patient-reported outcome measures (PROMs) at T1 and T3:

1. The State-Trait Anxiety Inventory–State version (STAI-S), a 20-item questionnaire measuring the transitory emotional condition of stress or tension perceived by the patient. Items could be scored on a 4-point scale ranging from ‘not at all’ (1) to ‘very much’ (4) [range 20–80].^17^
2. The three-item Cancer Worry Scale (CWS) measuring concerns about developing cancer again and the impact on daily activities.^18^–^20^ Higher scores mean more worries (range 3–12).
3. The 15-item Impact of Event Scale (IES) evaluating the extent to which patients suffer from life hazards, in this case having a melanoma, in terms of avoidance and intrusion.^21^,^22^ A higher score (range 0–75) corresponds to a higher level of stress response symptoms (SRS).
4. The RAND-36, a 36-item health-related quality-of-life questionnaire, of which the mental component (MCS) and physical component (PCS) summary scores were used. The summary scores are standardized, with a mean of 50 and a standard deviation of 10.^23^

Total melanoma-related hospital costs were calculated for 51 patients from a University Medical Center (Groningen) and 34 patients from a large teaching hospital (Isala Clinics, Zwolle) participating at T3 (representing 77.3% of participants). Costs per melanoma patient are considered largely comparable between hospitals as a consequence of the financing system in The Netherlands, which is a price-competitive reimbursement system. Costs per patient are calculated using diagnosis-treatment combinations (DBCs). DBCs are developed for a combination of interventions and treatments that belong to a certain diagnosis.^24^ These DBCs are fixed prices and are based on agreement between hospitals and health insurance companies. Costs taken into account included all follow-up visits and telephone consultations, as well as detection and treatment of recurrences. Expenses for GP consultations were not taken into account.

### Statistical Analysis

The power analysis performed has been described previously.^16^ Statistical analyses were performed using IBM SPSS statistics version 22 (IBM Corporation, Armonk, NY, USA). Patient characteristics were described, and comparisons between study groups were performed using independent *t* tests, Mann–Whitney *U* tests, Chi square tests, or Fisher’s exact tests, as appropriate. Repeated measures analyses of variance (ANOVAs) were conducted to examine differences between groups, time differences, and interaction effects in PROMs. Effect sizes (ESs) were computed to examine clinical relevance when a difference was found to be statistically significant. ES values ≥ 0.5 were considered large, those between 0.3 and 0.5 were considered moderate, and those < 0.3 were considered small.^25^ Cox proportional hazards models were computed to examine the effect of the group on recurrence-free survival (RFS) and disease-free survival (DFS).

*p* values < 0.05 were considered statistically significant.

## Results

Of the 207 patients who were eligible for inclusion, 27 refused participation (response rate = 87%),^16^ resulting in 180 participants being included at T1, of whom 87 were male (48%) and median age was 57 years (range 20–85). Patients were randomized into a conventional (CSG, *n* = 93) or experimental (ESG, *n* = 87) follow-up schedule group. No significant differences between study groups were found in sociodemographic or illness-related characteristics at T1.^16^

**Fig. 1:**
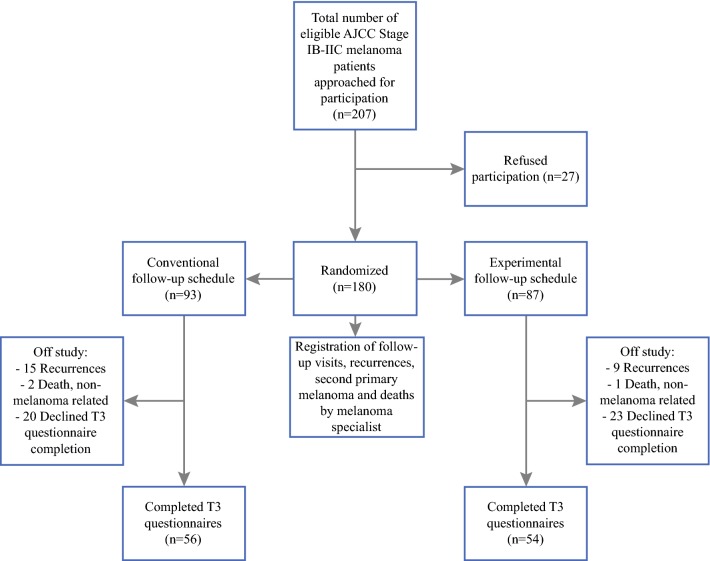
Flowchart of inclusion and randomization

At T3, 110 patients completed the questionnaire. Of the 70 patients who did not, 28 were excluded (recurrent disease, a second primary, or death) and 42 (23%) declined to complete T3 questionnaires (Fig. 1). No significant differences were found in sociodemographic and illness-related variables between T3 CSG and ESG participants (Table 2). T3 participants and those who dropped out were comparable in T1 sociodemographic and illness-related variables, as well as in mean PROMs scores (data not shown).

**Table 2:** Descriptives of sociodemographic and illness-related characteristics at T1, and follow-up-related questions at T3 of the 110 participants at T3, along with comparison between study groups (CSG, *n* = 56; ESG, *n* = 54) at T3

	Total [*n* = 110]	Conventional schedule [*n* = 56]	Experimental schedule [*n* = 54]	*p* value
*Characteristics at T1*				
Sex				0.181^c^
Female	56 (50.9)	25 (44.6)	31 (57.4)	
Male	54 (49.1)	31 (55.4)	23 (42.6)	
Age, years				0.161^d^
Mean ± SD (range)	56 ± 13 (24–81)	55 ± 14 (26–81)	58 ± 11 (24–78)	
Level of education^a^				0.312^c^
High	44 (40)	24 (42.9)	20 (37.0)	
Intermediate	44 (40)	24 (42.9)	20 (37.0)	
Low	22 (20)	8 (14.2)	14 (26.0)	
Relationship				0.189^c^
With partner	95 (86.4)	46 (82.1)	49 (90.7)	
Without partner	15 (13.6)	10 (17.9)	5 (9.3)	
Daily activities				0.257^c^
Employed for wages	59 (53.6)	33 (58.9)	26 (48.1)	
Not employed for wages	51 (46.4)	23 (41.1)	28 (51.9)	
Presence of comorbidities				0.053^c^
No	71 (64.5)	41 (73.2)	30 (55.6)	
Yes	39 (35.5)	15 (26.8)	24 (44.4)	
Primary melanoma site				0.463^c^
Lower extremity	32 (29.1)	20 (35.7)	12 (22.2)	
Upper extremity	21 (19.1)	9 (16.1)	12 (22.2)	
Trunk	46 (41.8)	22 (39.3)	24 (44.4)	
Head/neck	11 (10)	5 (8.9)	6 (11.2)	
Breslow thickness, mm				0.123^c^
< 1.0	8 (7.3)	1 (1.8)	7 (13.0)	
1.00–1.99	63 (57.3)	36 (64.3)	27 (50)	
2.00–3.99	31 (28.2)	15 (26.8)	16 (29.6)	
≥ 4.00	8 (7.3)	4 (7.1)	4 (7.4)	
Median (range)	1.7 (0.6–8.0)	1.6 (0.9–8.0)	1.7 (0.6–7.3)	
Ulceration				0.215^c^
No	85 (77.3)	46 (82.1)	39 (72.2)	
Yes	25 (22.7)	10 (17.9)	15 (27.8)	
AJCC classification				0.487^c^
Ib	65 (59.1)	34 (60.7)	31 (57.4)	
IIa	24 (21.8)	14 (25.0)	10 (18.5)	
IIb	15 (13.6)	5 (8.9)	10 (18.5)	
IIc	6 (5.5)	3 (5.4)	3 (5.6)	
*Follow*-*up*-*related questions at T3*				
Schedule satisfaction^b^				0.162^c^
No	9 (8.5)	7 (13)	2 (3.9)	
Yes	96 (91.5)	47 (87)	49 (96.1)	
Missing	5	2	3	
Reason for dissatisfaction^b^				0.444^e^
Wish for less visits	4 (44.4)	4 (57.1)		
Wish for more visits	5 (55.6)	3 (42.9)	2 (100)	
Adherence to follow-up schedule				**0.031**^**c**^
Less outpatient clinic visits than scheduled	11 (10)	7 (12.5)	4 (7.4)	
*1 visit less*	*6 (54.5)*	*3 (42.8)*	*3 (75)*	
*2 visits less*	*3 (27.3)*	*3 (42.8)*		
*3*–*4 visits less*	*2 (18.2)*	*1 (14.3)*	*1 (25)*	
Median (range)	1 (1–4)	2 (1–4)	1 (1–3)	0.466^f^
Conform schedule	74 (67)	42 (75)	32 (59.3)	
More outpatient clinic visits than scheduled	25 (23)	7 (12.5)	18 (33.3)	
+*1 extra visit*	*16 (64)*	*4 (57.1)*	*12 (66.7)*	
+*2 extra visits*	*5 (20)*	*1 (14.3)*	*4 (22.2)*	
+*3*–*5 extra visits*	*4 (16)*	*2 (28.6)*	*2 (11.1)*	
Median (range)	1 (1–5)	1 (1–4)	1 (1–5)	0.547^f^
Melanoma-related GP visits				0.439^c^
No	27 (24.5)	12 (21.4)	15 (27.8)	
Yes	83 (75.5)	44 (78.6)	39 (72.2)	
*Extra GP visits*				
+*1 visit*	*38 (45.8)*	*21 (47.7)*	*17 (43.6)*	
+*2 visits*	*29 (34.9)*	*17 (38.6)*	*12 (30.8)*	
+*3*–*5 visits*	*16 (19.3)*	*6 (13.6)*	*10 (25.7)*	
Median (range)	2 (1–5)	2 (1–5)	2 (1–5)	0.425^f^
Total (hospital + GP) extra visits	87 (79.1)	44 (78.6)	43 (79.6)	0.221^e^
+*1 extra visit*	*33 (37.9)*	*18 (40.9)*	*15 (34.9)*	
+*2 extra visits*	*25 (28.7)*	*16 (36.4)*	*9 (34.9)*	
+*3 extra visits*	*13 (14.9)*	*4 (9.1)*	*9 (20.9)*	
+*4 extra visits*	*10 (11.5)*	*3 (6.8)*	*7 (16.3)*	
+*5*–*7 extra visits*	*6 (6.9)*	*3 (6.8)*	*3 (7.0)*	
Frequency of self-inspection^b^				0.548^c^
Every week	18 (16.4)	8 (14.3)	10 (18.5)	
Every month	52 (47.3)	31 (55.4)	21 (38.9)	
Once every 3 months	26 (23.6)	11 (19.6)	15 (27.8)	
Less than every 3 months	12 (10.9)	5 (8.9)	7 (13.0)	
Never	2 (1.8)	1 (1.8)	1 (1.9)	
Hospital costs (3 years) [€]		*n* = *43*	*n* = *42*	
Follow-up visits		56,387.89	32,374.07	
Specialist		51,431.10	29,655.13	
NP		2538.10	1177.70	
Telephone consultation		2418.89	1541.24	
Diagnostics		12,344.22	6931.95	
Laboratory testing		322.76	6.00	
Ultrasonography		2044.96	819.96	
CT scan		775.89	872.00	
FDG-PET-CT scan		2771.42	1588.00	
Pathology/cytology		6429.19	3645.99	
Surgery		2450.00	2909.91	
Total costs (€)		71,182.11	42,215.93	
Costs per patient over 3 years [mean ± SD] (€)		1655.40 ± 921.3	1005.14 ± 745.05	***0.001***^***f***^

Data are expressed as *n* (%) unless otherwise specified

*CSG* Conventional Study Group, *ESG* Experimental Study Group, *AJCC* American Joint Committee on Cancer, *GP* general practitioner, *NP* nurse practitioner, *SD* standard deviation, *CT* computed tomography

^a^Highest level of education completed (high: vocational education, university; intermediate: secondary vocational education, high school; low: elementary school, low vocational education)

^b^Self-designed questions

^c^Chi square test

^d^Independent student *t* test

^e^Fisher’s exact test

^f^Mann–Whitney *U* test

Significant *p* values are shown in bold

No significant between-group differences in satisfaction with the follow-up schedule (*p* = 0.162) were found at T3, or in reason for dissatisfaction (*p* = 0.444). Adherence with the assigned follow-up schedule differed significantly between groups (*p* = 0.031). Significantly more ESG than CSG patients paid more visits to the medical specialist than scheduled. Of the patients who paid extra visits, 16 (64%) paid only one extra visit during the 3-year period. Medians for the number of fewer or extra visits did not differ between groups (*p* = 0.466 and *p* = 0.547, respectively) [Table 2]. Adherence to the assigned follow-up schedule and schedule satisfaction were not significantly related (Fisher’s exact test, *p* = 0.154). No significant difference was found between study groups in terms of melanoma-related GP visits (*p* = 0.439) or when combining extra visits to the medical specialist with the melanoma-related GP visits (*p* = 0.221). Of the 83 patients who paid extra GP visits, 46% did this only once (Table 2).

All patients reported performing self-inspection, except one CSG and one ESG patient. The frequency of self-inspection did not differ significantly between groups (*p* = 0.548) (Table 2).

### Patient-Reported Outcome Measures

Repeated measures ANOVA showed a significant between-group effect on the IES (*p* = 0.001) and the RAND-36 PCS (*p* = 0.02). ESG patients had significantly lower IES mean scores at T1 and T3, and had a significantly lower RAND-36 PCS score at T1 (*t* test; *p* = 0.006) but not at T3 (*t* test; *p* = 0.264). ESs were small. Over time, a significant decrease was found in mean scores on the CWS and IES, and an increase on the RAND-36 MCS and PCS scores (all *p* < 0.001). ESs were small. No significant interaction effects were found (Table 3).

**Table 3:** Descriptives of patient-reported outcome measures at T1 and T3, and repeated measures ANOVAs (CSG, *n* = 56; ESG, *n* = 54)

Questionnaire	Study group	T1 mean (SD)	T3 mean (SD)	Repeated measures ANOVA
STAI-S	Conventional	31.2 (8.3)	30.3 (9.4)	*F *= 0.2; *p *= 0.66 (group)
	Experimental	32.4 (8.1)	30.4 (7.9)	*F *= 3.3; *p* = 0.07 (time)
				*F *= 0.5; *p* = 0.48 (interaction)
CWS	Conventional	4.6 (1.5)	4.0 (1.8)	*F *= 0.3; *p *= 0.59 (group)
	Experimental	5.1 (2.2)	3.8 (1.0)	*F *= 22.5; ***p* < 0.001** (time), ES = 0.18
				*F *= 3.3; *p* = 0.07 (interaction)
IES	Conventional	23.3 (14.4)	14.0 (17.0)	*F *= 11.4; ***p *=0.001** (group), ES = 0.12
	Experimental	14.0 (13.2)	6.2 (8.5)	*F *= 31.5; ***p* < 0.001** (time), ES = 0.28
				*F *= 0.23; *p* = 0.64 (interaction)
RAND-36 MCS score	Conventional	49.6 (11.3)	53.5 (8.3)	*F *= 0.004; *p *= 0.95 (group)
	Experimental	48.6 (10.9)	54.3 (5.3)	*F *= 21.2; ***p* < 0.001** (time), ES = 0.16
				*F *= 0.81; *p* = 0.37 (interaction)
RAND-36 PCS score	Conventional	48.9 (9.0)	52.4 (8.4)	*F *= 5.4; ***p *= 0.02** (group), ES = 0.05
	Experimental	43.4 (11.3)	50.3 (10.6)	*F *= 29.8; ***p* < 0.001** (time), ES = 0.22
				*F *= 3.2; *p* = 0.08 (interaction)

*CSG* Conventional Study Group, *ESG* Experimental Study Group, *T1* at inclusion, shortly after diagnosis, *T3* 3 years later, *STAI*-*S* State-Trait Anxiety Inventory–State (range 20–80), *CWS* Cancer Worry Scale (range 3–12), *IES* Impact of Event Scale (range 15–75), *MCS* mental component summary of the RAND-36 (standardized mean of 50, SD of 10), *PCS* physical component summary of the RAND-36 (standardized mean of 50, SD of 10), *F* F-statistic, *ES* effect size, *SD* standard deviation, *ANOVAs* analyses of variance

Significant *p* values are shown in bold

### Melanoma Recurrences and Deaths During the 3-Year Follow-Up

At T3, 25 patients (13.9%) had been diagnosed with recurrent disease or a second primary—15 CSG patients (16.1%) and 10 ESG patients (12%) [*p* = 0.397]. The Cox proportional hazards model showed no significant difference between groups in RFS (hazard ratio [HR] 0.71 [0.32–1.58]; *p* = 0.400). Of the recurrences or second primaries, 15 were diagnosed within the first year^16^ and 10 (40%) were diagnosed between T1 and T3. No significant differences were found between groups in terms of locoregional and/or distant disease or second primaries (*p* = 0.457) at T3. Sixteen recurrences (66.7%) were detected by the patients themselves, and eight (33.3%) were detected by the medical specialist; study groups did not differ when considering who detected a recurrence (*p* = 0.204) (Table 4).

**Table 4:** Descriptives of recurrences and deaths, and comparison between groups (CSG, *n* = 93; ESG, *n* = 87)

Characteristics	Total [*n* = 180]	Conventional schedule [*n* = 93]	Experimental schedule [*n* = 87]	*p* value
Total recurrence or second primary during 3-year follow-up	25 (13.9)	15 (16.1)	10 (11.5)	0.397^a^
Median time, days (range)	406 (179–1040)	369 (203–1040)	423 (179–984)	0.618^b^
Specifically				0.457^c^
Locoregional recurrence	11 (45.8)	8 (53.3)	3 (33.3)	
Distant recurrence	6 (25)	3 (20)	3 (33.3)	
Locoregional + distant recurrence	2 (8.8)	2 (13.3)		
Second primary	5 (20.8)	2 (13.3)	3 (33.3)	
Missing	1		1	
Detection of recurrence or second primary				0.204^c^
Patient	16 (66.7)	11 (78.6)	5 (50)	
Specialist/NP	8 (33.3)	3 (21.4)	5 (50)	
Missing	1	1		
Died of melanoma during 3-years follow-up	13 (7.2)	6 (6.5)	7 (8)	0.777^a^
Median time, days (range)	780 (406–1169)	997 (415–1169)	712 (406–1017)	0.317^b^
Died of other cause	3 (1.7)	2 (2.2)	1 (1.1)	

Data are expressed as *n* (%) unless otherwise specified

*CSG* Conventional Study Group, *ESG* Experimental Study Group, *NP* Nurse Practitioner

^a^Chi square test

^b^Mann–Whitney *U* test

^c^Fisher’s exact test

Of the 25 patients who developed a recurrence or second primary during the 3-year period, 13 (7.2%) died of melanoma—6 CSG patients and 7 ESG patients (*p* = 0.777). A Cox proportional hazards model showed no significant difference in DFS between the groups (HR 1.24 [0.42–3.71]; *p* = 0.69).

### Cost Analysis

The total amount spent during the 3 years of follow-up was €71,182.11 for the 43 CSG patients and €42,215.93 for the 42 ESG patients. The mean amount spent per ESG patient was significantly lower than the amount spent per CSG patient (*p* = 0.001) [Table 2], and the total cost reduction was 39%. No significant differences were found in total costs between the two hospitals.

## Discussion

The current study showed that 3 years after diagnosis, patients assigned to the reduced stage-adjusted follow-up schedule (ESG) reported levels of anxiety, cancer worry, and mental health-related quality of life similar to those of patients assigned to the follow-up schedule as currently advised in the Dutch Melanoma guideline. Moreover, ESG patients reported significantly lower levels of SRS. Additionally, over the 3-year period, recurrences and second primary melanomas were detected within a comparable time period in both groups, and the number of patients dying from melanoma and time until death were equal. Lastly, a reduced stage-adjusted follow-up schedule resulted in a 39% cost reduction in the ESG. These results support our hypotheses of no differences in PROMs, recurrences and deaths between study groups, and lower costs in the experimental group. They suggest that a less-frequent follow-up schedule than is currently recommended in the Dutch Melanoma guideline does not negatively affect melanoma patients in terms of quality of life, or in terms of the time until, and the number of patients diagnosed with, recurrent disease and/or dying from melanoma. Moreover, costs would be decreased.

The present 3-year results are in line with, and thus support, the 1-year MELFO results.^16^ As at 1 year, at 3 years ESG patients reported suffering less from SRS. The literature suggests that 50% of patients report having high anxiety before and during outpatient clinic visits.^26^ Our findings suggest that a less-frequent follow-up schedule, thus less exposure to such anxious events, is beneficial in the short- and long-term because it induces fewer SRS. However, the ES of the between-group difference in SRS at 3 years is small, indicating that the difference is clinically not relevant, while the ES at 1 year was moderately large. This suggests that the difference in SRS between groups becomes clinically irrelevant over time.

As after 1 year,^16^ after 3 years most ESG and CSG patients were satisfied with the assigned schedule. This implies that patients were content with the follow-up schedule suggested by their doctor, be it conventional or reduced. However, four-fifths of patients paid fewer or more melanoma-related visits, indicating that patients seek or decline medical attention when they judge it to be necessary or not.

A significantly higher percentage of ESG patients than CSG patients paid extra visits to the medical specialist than scheduled. However, of those who paid extra visits, two-thirds of the ESG patients and more than half of the CSG patients paid only one extra visit during the 3-year study period. Therefore, it seems unlikely that extra visits will have affected the 3-year results of the current study in terms of experienced quality of life or detection of a recurrence or second primary. Additionally, three-quarters of patients paid extra visits to the GP, with, again, almost half (in both groups) paying only one extra visit in the 3 years of follow-up. The reason for these extra visits may be increased awareness of suspicious lesions, possibly resulting from effective education on self-inspection.^4^,^11^–^14^,^26^–^29^

The current 3-year results show that the number of recurrences and second primary melanomas, and the time until detection for patients with pathological sentinel node staged AJCC stage IB–IIC, was independent of the assigned follow-up schedule, which is in line with the 1-year MELFO results.^16^ Almost two-thirds of the recurrences were detected within the first year after diagnosis, and two-fifths were detected between 1 and 3 years after diagnosis. This is conform literature, showing that the highest proportion of melanoma recurrences and second primaries is detected during the first year of follow-up, and that the proportion declines over the following years.^4^,^9^,^13^,^14^

The present study showed that almost two-thirds of patients detected a recurrence themselves, which, again, is conform literature.^13^,^14^,^26^ No differences were found between study groups, which suggests that the patient information provided was comparable between study groups.

Overall, the 3-year recurrence rate in the present study was 13.9%, which is comparable with recent literature reporting a rate of 14.7%.^4^ However, it is slightly lower than the 19% reported in a retrospective study including AJCC stage IA–IIC melanoma patients and with a much longer follow-up period (range 0–26.6 years).^9^ An explanation for the higher percentage found in that study may be the inclusion of patients who had not been sentinel node staged, resulting in underestimation of the disease stage and, consequently, the risk of recurrence.^30^ Second, although most recurrences are detected within 3 years after diagnosis, some patients do develop a recurrence after 3 years.^9^

Thirteen patients in the current study died of their melanoma within 3 years after diagnosis (7.2%), with no difference between the follow-up schedule groups. This is slightly lower than the 8.2% reported in another prospective study; however, that study followed patients until 4 years after diagnosis.^4^

There is no consensus in the literature with respect to performing routine additional laboratory testing (biomarkers LDH, S-100B) and imaging (ultrasonography, chest x-ray, positron emission tomography [PET], magnetic resonance imaging [MRI]) during follow-up in pathological sentinel node staged AJCC IB–II melanoma patients, even in high-risk melanoma patients (stage IIB/C), with some being in favor and others not.^31^ The argument of those who are against is that three-quarters of first recurrences are detected by patients themselves. They recommend to perform additional testing and imaging only when (distant) recurrent disease is suspected.^7^,^13^,^14^,^32^ For patients with local, regional, or metastatic disease, various treatment options are available, namely systemic treatment options such as BRAF/MEK inhibitors, and immunologic strategies with CTLA4, PD-1/PD-L1 antagonists that result in significantly improved survival rates^33^

After 3 years, a less-frequent follow-up schedule resulted in a considerable cost reduction (39%), as found after 1 year.^16^ Healthcare costs are high, financially burdening healthcare systems and societies. The present study shows that a reduced stage-adjusted follow-up schedule is cost effective and is safe for patients. Additionally, less-frequent follow-up will save healthcare providers’ time, now and in the future, considering the increasing melanoma prevalence. Increasingly, in The Netherlands, melanoma-trained nurse practitioners provide follow-up and specific patient melanoma (E-health) education in dedicated melanoma clinics.^29^ This will further reduce costs in melanoma care.

The current study has some limitations. First, 23% of patients declined to participate at 3 years after diagnosis; however, this percentage is lower than the dropout rate in another prospective study in melanoma patients.^4^ Fortunately, no differences were found in baseline characteristics and PROMs between patients who did and did not complete T3 questionnaires. Second, power analysis showed that 89 patients per group were needed. We commenced with 93 patients in the CGS group and 87 patients in the ESG group. Due to the dropout rate over 3 years, the number of patients analyzed at T3 is lower than envisaged. However, no differences in sociodemographic and illness-related variables were found between participants in the two study groups at either T1^16^ or T3. Third, due to the small sample size, some analyses performed should be interpreted carefully.

## Conclusion

The 3-year results of the MELFO study seem to support the notion that a reduced stage-adjusted follow-up schedule is an appropriate, safe, and cost-effective alternative for pathological, sentinel node staged, AJCC stage IB–IIC melanoma patients, in terms of quality of life, recurrences, deaths, and financial costs, to the follow-up regimen as advised in the current melanoma guideline.
